# Implementation of Calcium and Vitamin D Supplementation in Glucocorticosteroid-Induced Osteoporosis Prevention Guidelines—Insights from Rheumatologists

**DOI:** 10.5041/RMMJ.10497

**Published:** 2023-04-30

**Authors:** Tal Gazitt, Joy Feld, Devy Zisman

**Affiliations:** 1 Rheumatology Unit, Carmel Medical Center, Haifa, Israel; 2 The Ruth and Bruce Rappaport Faculty of Medicine, Technion–Israel Institute of Technology, Haifa, Israel

**Keywords:** Education, glucocorticosteroid-induced osteoporosis, guideline implementation, prevention

## Abstract

Glucocorticosteroid-induced osteoporosis (GIO) is the most common cause of secondary osteoporosis but is underdiagnosed and undertreated. Our aim in this communication is to review the literature on the implementation of current GIO prevention practices such as calcium and vitamin D supplementation with emphasis on the rheumatologists’ perspective relating to the need for development of novel GIO educational prevention measures.

## INTRODUCTION

Glucocorticosteroid-induced osteoporosis (GIO) secondary to chronic glucocorticosteroid (GC) use is the most common cause of secondary osteoporosis. However, it is underdiagnosed and undertreated.^1^ Continuous GC therapy is associated with increased fracture risk within 3–6 months of initiation and is dose-related, with vertebral fractures being the most common.^1^ Estimates indicate that GC-induced fractures occur in 30%–50% of patients receiving long-term GC therapy. Furthermore, these fractures may be asymptomatic and may be the first sign of osteoporosis,^2^ with the highest relative risk for vertebral fractures ranging from 2.60 (95% confidence interval [CI] 2.31–2.92) to 2.86 (95% CI 2.56–3.16).^3^

To determine whether or not additional GIO prevention medications should be prescribed, the 2017 American College of Rheumatology (ACR) Guideline for the Prevention and Treatment of GIO recommended using the Fracture Risk Assessment Tool (FRAX®) score to determine the 10-year probability of major osteoporotic fracture risk in patients, stratified as low (<10% risk), moderate (10%–19% risk), or high (≥20%).^4^ This recommendation is being reformulated for the updated 2022 ACR Guideline but has not yet been finalized and published, although a summary of changes is available on the internet.^5^ These cut-off points can be used to weigh potential benefits versus harm when considering osteoporosis therapy, with a strong recommendation for oral bisphosphonates to patients receiving long-term GC therapy and at high risk for fracture. For GC-treated adults at moderate to high risk of fracture, oral or intravenous bisphosphonates, parathyroid hormone/parathyroid hormone-related protein (PTH/PTHrP) analogs, and denosumab are preferred agents depending on patient and physician preferences. The new guideline advises that selective estrogen receptor modulators (SERMs) and the newly added anti-sclerostin antibody romosozumab may be used in selected patients after careful consideration of potential harm including thrombosis, stroke, and cardiovascular events.

As an initial pharmacologic step for GIO prevention, most professional guidelines^4^,^6^ recommend calcium and vitamin D (Ca/VitD) supplementation for patients treated with 5–7.5 mg daily prednisone (or its equivalent) for 1–3 months. The 2017 ACR GIO prevention guideline also recommends that in patients starting on long-term GC therapy of 2.5 mg/day or more for at least 3 months, the calcium (Ca) and vitamin D (VitD) intakes should be optimized (1000–1200 mg/day and 600–800 IU/day, respectively).^4^ Along these lines, the European League Against Rheumatism (EULAR) notes in recommendation #9 that Ca/VitD supplementation is important for osteoporotic fracture management in patients over 50 years of age.^7^ However, actual rates of GIO prophylaxis using Ca/VitD supplementation are low, ranging from 6%–11% in the United Kingdom (UK) in 1995–1996^8^ to higher rates (30%–62%) in more recent studies.^9^,^10^

Our aim in this communication was to evaluate the current status of educational interventions aimed at increasing awareness of, and implementation of, the basic GIO prevention guidelines using Ca/VitD supplementation among physicians and patients from 2000 onward.

## METHODS

We performed a PubMed literature search for articles on educational interventions regarding GIO prevention with the following key words: “glucocorticosteroid-induced osteoporosis (GIO) prevention,” “GIO prevention guideline adherence,” “GIO prevention education,” and “GIO education intervention.” We noted articles discussing implementation of Ca/VitD recommendations as well as articles proposing interventions to increase implementation of supplementation recommendations from 2000 to 2018.

## RESULTS

In our literature search on current educational efforts aimed at increasing awareness of and adherence to Ca/VitD supplementation during 2000–2018, we found 2–13 articles under each search term used. Only a few papers were found related to Ca/VitD supplementation adherence in real-life practice, and only 3 studies focused on actual educational interventions regarding adherence to increasing Ca/VitD supplementation.^11^–^13^ No studies were found that focused on Ca/VitD supplementation in the transition of care from the inpatient to the outpatient setting.

In order to fill this gap in the literature, we initiated a study in 2018 on Ca/VitD supplementation as initial GIO prevention measure in hospitalized patients anticipated to receive long-term GC treatment.^14^ We showed that although rheumatologists recommended Ca/VitD supplementation in 79.2% of relevant hospitalizations, it was only prescribed upon discharge in 44.3% of relevant hospitalizations in internal medicine wards. An attempt we made as rheumatologists to increase awareness of inpatient healthcare providers in internal medicine wards regarding the indications for and importance of Ca/ VitD supplementation through standardized GIO prevention lectures had no impact on discharge prescriptions.^14^

Similar to the study we conducted, several of the retrospective studies reviewed herein showed poor adherence to Ca/VitD supplementation as the initial pharmacologic GIO prevention measure.^9^,^14^–^16^ Aagaard et al. examined the types GIO prevention measures implemented among 215 outpatients of San Francisco General Hospital who had been taking at least 5 mg/d of prednisone for a minimum of one month.^9^ The study also looked at patient and provider characteristics associated with these measures. They found 124 of the 215 outpatients (58%) had been treated with the following GIO prophylactic measures: Ca alone (42%), VitD alone (37%), Ca/VitD (30%), hormone replacement therapy (HRT) (57%), or bisphosphonates (4%). They also noted that patients receiving GIO prophylaxis were older females (especially post-menopausal) with more comorbidities and polypharmacy, as well as patients followed in rheumatology clinics. Similar results for low Ca/VitD supplementation rates were found in a cross-sectional study by Guzman-Clark et al. on 100 Veterans Administration patients on 5 mg/day or more of prednisone for at least 3 months (Ca, 32%; multivitamin, 19%; VitD, 12%).^15^ Yood et al.’s retrospective study on 224 outpatients who received at least one oral GC prescription per quarter also found that only 18%–36% of patients received Ca/VitD supplementation when GC were prescribed by primary care physicians, internists, pulmonologists, or other subspecialists but increased to 64% when GC were prescribed by rheumatologists.^16^ They also found that any GIO prophylactic intervention was more likely to be given to women than to men (76% versus 44%, respectively, prevalence odds ratio [OR] 4.02; 95% CI 2.19–7.43), and increased likelihood was also found in rheumatology patients (62/69, 90%; OR 6.70; 95% CI 2.78–16.19).

Several studies to date have examined patient and provider-related barriers to GIO prevention, pointing to the lack of efficacy of in-person counseling and educational lectures to increase adherence to GIO prevention measures. For instance, Blalock et al.’s cross-sectional study evaluated patient recall of osteoporosis prevention counseling among 227 rheumatology patients who had filled at least two prednisone prescriptions within the previous 6 months (91.2% with previous diagnosis of rheumatoid arthritis and 15.4% with a previous diagnosis of osteoporosis).^17^ They showed that while 97.4% of patients had heard of osteoporosis, only 50.2% recalled being told they were at increased risk of osteoporosis, and only 36.3% recalled receiving osteoporosis prevention counseling. Where such counseling was given, 73.2% of patients recalled being informed about Ca supplementation, but had poor recollection of other GIO prevention recommendations. Interestingly, in actuality, Ca supplementation at the appropriate recommended doses was given only to 51.1% of the 227 study participants, and VitD was prescribed to only 35.2% of cases. Patients were more likely to be counseled regarding GIO prevention if they were white, with a previous osteoporosis diagnosis, and with some college education.

In an attempt to examine barriers to physician implementation of GIO prophylactic measures, Guzman-Clark et al.^15^ included focus groups to examine factors serving as barriers to physician implementation of GIO prophylactic measures. They found that only 4/23 (17.4%) of providers correctly identified dose and duration of GC use requiring GIO prophylaxis based on the then-current 2001 ACR guideline. Common GIO management barriers cited by physicians were lack of physician knowledge, limited time in clinic visit, and patient non-adherence. Suggestions for improvement cited by physicians included patient educational handouts, clinician pocket guide with clinical guidelines, and pharmacy-based computerized clinical reminders.^15^

Extending the above studies regarding educational interventions among osteoporosis patients, a recent Australian study proposed an actual interventional measure aimed at increasing patient understanding and recollection of osteoporosis prevention recommendations.^9^ Under the guidance of a community partnership research team,^15^ osteoporosis patients built an oversized jigsaw puzzle made of 6 puzzle pieces with messages on the back about the recommended guidelines on osteoporosis prevention.^11^ These messages were used to prompt audience recall following presentation of the information so that a strong puzzle made of well-fitting puzzle pieces (representing strong bone health), could be built from these prompts. In this intervention, six additional oversized jigsaw puzzle pieces, designed not to fit into the puzzle, were made by the patients with erroneous messages and myths regarding osteoporosis prevention on the back (for example, “osteoporosis does not affect men”). Regrettably, this report did not evaluate data on the success of this strategy in actually increasing guideline adherence.

When searching for educational efforts aimed at increasing awareness of the importance of basic GIO prevention methods among providers, we found only one other interventional study, which was performed in an academic-based rheumatology practice.^12^ This study involved 373 rheumatoid arthritis patients on chronic GC treatment and was aimed at increasing adherence to GIO prevention measures using (1) a lecture and discussion regarding optimal management of GIO; (2) confidential doctor-specific audit on GIO management; and (3) mailed reminders including concise pharmacologic recommendations. Similar to our study, this three-pronged interventional measure had no impact on GIO prevention measures, nor on bone mineral density (BMD) testing and pharmacologic therapy.^12^ Recently, more effort has been placed into utilizing computerized systems to help increase adherence to osteoporosis prevention and treatment guidelines. For instance, a 2018 audit^13^ on implementation of the British National Osteoporosis Guidelines Group (NOGG) 2017 guidelines^6^ utilized a computerized healthcare intelligence tool able to access primary care clinical data on 5,496 patient subscribers. The audit singled out patients prescribed GC at least three times during the last calendar year and classified this patient population as at high, intermediate, or low risk of osteoporosis fracture based on the frequency and dose of GC exposure and FRAX® score, if available. Automated letters were sent to patients meeting the defined thresholds: high-risk patients received an alendronate prescription with an explanation; intermediate-risk patients received a BMD testing referral; and low-risk patients received an educational leaflet on lifestyle changes. Following implementation of this algorithmic tool, adherence to bisphosphonate treatment for qualifying patients improved from 25% to 92% within a 6-month period.^13^ One limitation of this computerized tool was its inclusion of patients with chronic kidney disease or patients with upcoming dental work who would normally not be prescribed bisphosphonates; however, such computerized algorithms may be improved once instituted and could play a role in GIO prevention.

## DISCUSSION

While recent data on VitD supplementation has not supported its use to reduce risk of falls^18^ or fractures^19^ in generally healthy midlife and older adults without known osteoporosis, with other data showing positive effects of VitD supplementation such as reduced risk of autoimmune diseases in this population,^20^ Ca/Vit D supplementation is still being currently recommended by most guidelines as a primary preventive measure for GIO. In line with the results of our study and older studies on GIO, current literature shows that adherence to Ca/VitD supplementation in GIO prevention guidelines is low. However, as frequent prescribers of GC, rheumatologists were found both in our study and in our literature search to play an important role in implementing osteoporosis prevention measures due to their increased awareness of GIO prevention measures.^14^–^17^,^21^–^23^

Unfortunately, thus far, only few studies were conducted on increasing physician awareness of the need for GIO prevention measures, as we have done in our study on Ca/VitD supplementation as a first step in reducing GIO. As shown by the NOGG study from the United Kingdom and with the increase in use of electronic medical records and computerized algorithms for physicians in many aspects of medicine,^23^ it makes sense to employ these technologies in the field of preventative health care; GIO prevention should be included in these computerized management protocols given the increased morbidity associated with this condition and the lack of physician awareness of interventional measures.

## CONCLUSION

In conclusion, our study on the adherence to Ca/ VitD supplementation as an initial pharmacologic step for GIO prevention in hospital-discharged patients^14^ as well as this literature search on this subject found low adherence to Ca/VitD supplementation among non-rheumatology providers. Similar to other studies, an attempt at traditional educational intervention had no impact on adherence to Ca/VitD supplementation among non-rheumatology providers. Literature on physician practices related to GIO prevention measures suggests that rheumatologists, who generally have a better awareness of these recommendations, could play an important role in leading changes to improve GIO prevention by developing and implementing non-traditional educational strategies to educate physicians and patients about GIO prevention. Such strategies could be of significant value for patients newly started on GC therapy, especially at the transitional point of care from the hospital to the community.

## Abbreviations

BMD: bone mineral density
Ca: calcium
Ca/VitD: calcium and vitamin D
EULAR: European League Against Rheumatism
FRAX: Fracture Risk Assessment Tool
GC: glucocorticosteroid
GIO: glucocorticosteroid-induced osteoporosis
NOGG: National Osteoporosis Guidelines Group
PTH/PTHrP: parathyroid hormone/parathyroid hormone-related protein
SERMs: selective estrogen receptor modulators
UK: United Kingdom
VitD: vitamin D
